# Ribociclib enhances infigratinib‐induced cancer cell differentiation and delays resistance in FGFR‐driven hepatocellular carcinoma

**DOI:** 10.1111/liv.14728

**Published:** 2020-11-23

**Authors:** Aldo Prawira, Thi Bich Uyen Le, Thanh Chung Vu, Hung Huynh

**Affiliations:** ^1^ Laboratory of Molecular Endocrinology Division of Molecular and Cellular Research National Cancer Centre Singapore

**Keywords:** differentiation therapy, drug resistance, FGFR inhibitor, liver cancer

## Abstract

**Background & Aims:**

Infigratinib is a pan‐FGFR (fibroblast growth factor receptor) inhibitor that has shown encouraging activity in FGFR‐dependent hepatocellular carcinoma (HCC) models. However, long‐term treatment results in the emergence of resistant colonies. We sought to understand the mechanisms behind infigratinib‐induced tumour cell differentiation and resistance and to explore the potential of adding the CDK4/6 inhibitor ribociclib to prolong cell differentiation.

**Methods:**

Nine high and three low FGFR1‐3‐expressing HCC patient‐derived xenograft (PDX) tumours were subcutaneously implanted into SCID mice and subsequently treated with either infigratinib alone or in combination with ribociclib. Tumour tissues were then subjected to immunohistochemistry to assess cell differentiation, as indicated by the cytoplasmic‐to‐nuclear ratio and markers such as CYP3A4, HNF4α and albumin. Western blot analyses were performed to investigate the signalling pathways involved.

**Results:**

Infigratinib induced cell differentiation in FGFR1‐3‐dependent HCC PDX models, as indicated by an increase in the cytoplasmic/nuclear ratio and an increase in CYP3A4, HNF4α and albumin. Resistant colonies emerged in long‐term treatment, characterised by a reversal of differentiated cell morphology, a reduction in the cytoplasmic‐to‐nuclear ratio and a loss of differentiation markers. Western blot analyses identified an increase in the CDK4/Cdc2/Rb pathway. The addition of ribociclib effectively blocked this pathway and reversed resistance to infigratinib, resulting in prolonged cell differentiation and growth inhibition.

**Conclusions:**

Our findings demonstrate that the combined inhibition of FGFR/CDK4/6 pathways is highly effective in providing long‐lasting tumour growth inhibition and cell differentiation and reducing drug resistance. Therefore, further clinical investigations in patients with FGFR1‐3‐dependant HCC are warranted.

**Abbreviations**
ALB: albumin
CDK: cyclin‐dependent kinase
CSC: cancer stem cell
CYP3A4: cytochrome P450 3A4
FGF: fibroblast growth factor
FGFR: fibroblast growth factor receptor
HCC: hepatocellular carcinoma
HNF4α: hepatocyte nuclear factor 4 alpha
PDX: patient‐derived xenograft
TPM: transcripts per kilobase million

**Key points**

Infigratinib is an FGFR inhibitor that is highly effective against FGFR‐dependant hepatocellular carcinoma. However, long‐term treatment with infigratinib resulted in the emergence of drug‐resistant tumour characterised by an increase in CDK4/Cdc2/Rb pathway. The addition of clinically approved ribociclib overcomes infigratinib resistance, delays tumour growth and potentially translating to prolonged patient survival.

## 1 INTRODUCTION

In 2018, liver cancer collectively ranked as the sixth most prevalent neoplasm and the fourth leading cause of cancer‐related mortality worldwide.^1^ These cases are predominantly primary hepatocellular carcinoma (HCC), making up to 85% of the total diagnoses. To date, prognosis remains grim, with a recent estimate indicating a 5‐year overall survival of less than 12%.^1^ Surgical hepatic resection is effective against localised tumours; however, only less than 30% of patients are eligible for such treatment modality, often because multiple lesions are present at the time of diagnosis. Following its approval in 2007, sorafenib has remained the only first‐line therapy for advanced HCC, albeit with the modest survival benefit of only several months.^2^ Since then, numerous treatments have failed to improve treatment outcome^3^, ^4^ with only lenvatinib showing superior overall survival (OS), making it the next approved first‐line therapy.^5^ More recently, a clinical trial on the combination of atezolizumab and bevacizumab on advanced HCC showed significant improvement in overall and progression‐free survival when compared against sorafenib, rendering it the new benchmark for first‐line therapy.^6^ Yet, given the heterogeneity of HCC, there is still a need to improve treatment options for a certain subset of patients.

Increasing evidence suggests that cancers arise from a distinct population of tumour‐initiating cells or cancer stem cells (CSCs). First identified in acute myeloid leukaemia (AML),^7^ CSCs have also been described in solid cancers, including HCC.^8^, ^9^, ^10^ CSCs represent a small subset of poorly differentiated cells that display characteristics such as self‐renewal, higher tumorigenicity and resistance to treatments. In the context of HCC, MYC inactivation resulted in the differentiation and eventual death of most of the tumour cells, but some appeared to have retained the potential to differentiate into multiple hepatic lineages.^11^ This subpopulation of cells may exist as dormant CSCs.^12^, ^13^, ^14^ Clinically, it is frequently observed that, after therapy, tumours exist in a latent state but are still capable of reverting back to a neoplastic state.^15^, ^16^ The fibroblast growth factor (FGF) and its receptor (FGFR) family play an integral role in CSC initiation, partly through the activation of the Akt/β‐catenin pathway.^17^ Ligands binding to FGFRs promote cancer cell proliferation via MAPK/ERK signalling and increase survival through the PI3K/Akt pathway.^18^ Additionally, the FGF/FGFR axes have also been implicated in the recruitment of stromal cells, which is important for HCC progression.^19^

Treating malignant tumours through ‘differentiation therapy’ has been an attractive concept, but clinical development of differentiation‐inducing agents to treat malignant tumours, especially solid tumours, has been limited to date.^20^, ^21^, ^22^ This differentiation‐based approach does not eradicate cancer cells but rather reverses the aggressive phenotype to a more benign state or at the very least a lower grade of aggressiveness, rendering it more amenable to conventional therapeutic approaches.

There have been a number of studies exploring pharmacological agents to drive CSCs into dormant, terminally differentiated cells. In acute promyelocytic leukaemia, all‐trans retinoic acid (ATRA) has been proven to be highly successful in initiating CSC differentiation into more short‐lived granulocytes,^21^ ultimately resulting in complete remission in up to 90% of patients.^23^ However, similar evidence is lacking in HCC, with observations limited to only a few experimental studies.^24^, ^25^, ^26^, ^27^ Importantly, evidence on whether HCC CSC truly differentiates into cells that are less neoplastic is wanting, rendering it difficult to ascertain terminal differentiation. For instance most studies did not examine histological markers of mature hepatocytes, such as the hepatocyte nuclear factor‐4α (HNF4α), cytokeratin‐18 (CK‐18), cytochrome P450 3A4 (CYP3A4) or albumin.^28^, ^29^, ^30^

Our previous study has established the ability of infigratinib to inhibit tumour growth, normalise tumour vascularisation, reduce intratumoural hypoxia and impair invasion and metastasis in human HCC PDX models.^31^ Given the significance of FGFR in cell growth and differentiation, we explore the potential of targeting FGFR‐dependent HCC with infigratinib to induce differentiation, as evidenced by an increase in the cytoplasmic/nuclear ratio and immunocytochemical differentiation markers, as well as a reduction in proliferation. This study investigates infigratinib as a single agent and in combination with the CDK4/6 inhibitor ribociclib for longer‐lasting differentiation, attenuation of CSC characteristics and prevention of the emergence of dedifferentiated drug‐resistant colonies.

## 2 MATERIALS AND METHODS

### 2.1 Reagents

Ribociclib (S7440) and Infigratinib (S2183) were purchased from Selleck Chemicals and were dissolved in 21% Captisol and 30% PEG300 solution (vehicle) for oral administration. Antibodies against FGFR1 (#9740), FGFR3 (#4574), FGFR4 (#8562), AKT (#9272), Rb (#9313), cyclin B1 (#4138), Cdc25C (#4688), Cyclin D1 (#2978), Cyclin E2 (#4132), survivin (#2803), Sox9 (#82630), cleaved PARP (#5625), β catenin (#8480), α‐tubulin (#2144) and phosphorylation‐specific antibodies against AKT Ser473 (#9271), FRS2‐α Tyr439 (#3861), Rb Ser807/811 (#9308), Histone 3 Ser10 (#9701), Cdc2 Tyr15 (#9111) and Erk1/2 Thr202/Tyr204 (#4370) were obtained from Cell Signaling Technology. Antibodies against CYP3A4 (ab124921) and HNF4‐α (ab201460) were from Abcam. The antibodies against FGFR2 (#sc‐122), Erk1/2 (#sc‐94), FRS2‐α (#sc‐17841), GAPDH (sc‐166545) and Cdk2 Thr14/Tyr15 (#sc‐28435‐R) were from Santa Cruz Biotechnology Inc. Anti‐mouse CD31 antibody (#2502) was from BioLegend. Anti‐albumin (#SAB4200711; clone HAS‐11) antibody was purchased from Sigma‐Aldrich.

### 2.2 In vivo studies

This study was approved by the SingHealth Institutional Animal Care and Use Committee (IACUC number: 2015/SHS/1020) and the National Cancer Centre Singapore Ethics Board. All animals received humane care according to the criteria outlined in the ‘Guide for the Care and Use of Laboratory Animals’ published by the National Institutes of Health.^32^ Male C.B‐17 SCID mice aged 9‐10 weeks and weighing 23‐25 g (InVivos Pte. Ltd.) were provided with sterilised food and water and housed in negative pressure isolators set at 23°C and 43% humidity with 12‐hour light/dark cycles. Nine high (HCC13‐0109, HCC01‐0909, HCC26‐0808A, HCC21‐0208, HCC29‐1104, HCC09‐0913, HCC25‐0705A, HCC06‐0606, HCC13‐0212) and three very low (HCC26‐1004, HCC2‐1318, HCC10‐0505) FGFR1‐4‐expressing HCC patient‐derived xenograft (PDX) tumours were subcutaneously implanted into 10 mice per treatment arm, as previously described.^33^ All drug administrations were initiated when tumour size reached approximately 170‐250 mm^3^. For Western blot analyses, mice were treated for 4 days and dosing started when tumour size reached 800 mm^3^.

To determine the optimum dose for the induction of differentiation, mice bearing tumour xenografts were orally treated with vehicle or 10, 15 or 20 mg/kg infigratinib once daily for 4 days. Subsequently, the optimal dose was used to study the antitumour activity of infigratinib in tumour‐bearing mice. To investigate the cell differentiation‐inducing effects of infigratinib, mice were treated daily with either vehicle or 15 mg/kg infigratinib for 12‐21 days. For the drug combination study, mice were randomised into four groups with 10 mice per treatment arm and subsequently orally administered once daily with (a) 200 µL vehicle, (b) 15 mg/kg infigratinib, (c) 75 mg/kg ribociclib or (d) a combination of 15 mg/kg infigratinib and 75 mg/kg ribociclib.

To generate infigratinib‐resistant PDX models, HCC01‐0909, HCC21‐0208, HCC26‐0808A and HCC06‐0606 PDX lines were subcutaneously implanted, and mice were repeatedly administered a daily dose of 15 mg/kg infigratinib. Mice were sacrificed once tumours reached approximately 1800 mm^3^. Infigratinib‐resistant tumours were collected, processed and re‐implanted into treatment‐naïve SCID mice. The infigratinib treatment cycle was then repeated for up to nine passages to maintain resistance. To detect the emergence of infigratinib‐resistant colonies that occurred during the early stages of acquired resistance, tumour tissues were collected when disease progression was observed.

Tumour volume was measured and calculated over the course of the study, as previously described.^33^ The efficacy of infigratinib alone or the infigratinib/ribociclib combination was determined with the T/C ratio, where T and C are the median weights of drug‐treated and vehicle‐treated tumours at the end of treatment respectively. T/C ratios of less than 0.42 are considered active, as determined according to the criteria of the Drug Evaluation Branch of the Division of Cancer Treatment, National Cancer Institute.^34^

### 2.3 Western blot analysis

Tumours from vehicle‐ and drug‐treated mice were homogenised in buffer containing 50 mmol/L Tris‐HCl pH 7.4, 150 mmol/L NaCl, 0.5% NP‐40, 1 mmol/L EDTA and 25 mmol/L NaF, supplemented with proteinase inhibitors and 10 mmol/L Na_3_VO_4_. Proteins (80 µg) were resolved by SDS‐PAGE and transferred onto a nitrocellulose membrane. Blots were probed with the indicated primary antibodies, followed by incubation with horseradish peroxidase‐conjugated secondary antibodies, as previously described.^31^ Subsequently, blots were visualised with an enhanced chemiluminescent detection system (Amersham, Pharmacia Biotech).

### 2.4 Immunohistochemistry

Tumours were fixed in 10% formalin overnight and subsequently embedded in paraffin blocks. Tissue sections (5 µm) were immunostained with antibodies against CD31, p‐Histone H3 Ser10 and cleaved PARP to assess microvessel density, cell proliferation and apoptosis respectively. Epitopes were visualised with SignalStain Boost IHC Detection Reagent (Cell Signaling Technology). To detect markers of hepatocyte differentiation, tissue sections were stained with antibodies against CYP3A4, HNF4α and albumin. Five random images were taken on an Olympus BX60 microscope (Olympus) and subsequently analysed. Image contrast and brightness were uniformly adjusted for clarity.

For tumour hypoxia staining, tumour‐bearing mice were intraperitoneally injected with 60 mg/kg pimonidazole hydrochloride 1 hour prior to tumour collection. To identify hypoxic regions, tumour sections were stained with HypoxyProbe Plus Kit HP2 (HypoxyProbe Inc) according to the manufacturer's protocol.

### 2.5 Quantification of cytoplasmic‐to‐nuclear ratio

Tissue sections were immunostained with antibodies against β‐catenin to define the cytoplasmic boundary and subsequently counterstained with haematoxylin to visualise nuclei. The areas of the nucleus and cytoplasm were measured using [(Length) × (Width^2^) × (π/6)], and the cytoplasmic‐to‐nuclear area ratio was then calculated. An increase in the ratio indicates differentiated cells. Five random fields at a magnification of ×400 from each treatment were captured with an Olympus BX60 microscope (Olympus), with at least 20 cells per field of view analysed.

### 2.6 Statistical analysis

The mean tumour volume between treatments was compared using two‐way ANOVA. Student's *t* test was used to compare the mean number of cells per field, tumour weight at sacrifice and mice body weight at the end of the treatment cycle. The Shapiro‐Wilk normality test was performed prior to Student's *t* test analysis. Results were considered statistically significant at *P* < .05. Graphs were generated using GraphPad Prism 8 (v. 8.2.1). All authors had access to the study data and had reviewed and approved the final manuscript.

## 3 RESULTS

### 3.1 The effect of infigratinib on the FGFR pathway, tumour growth and its clinical potential

**FIGURE 1:**
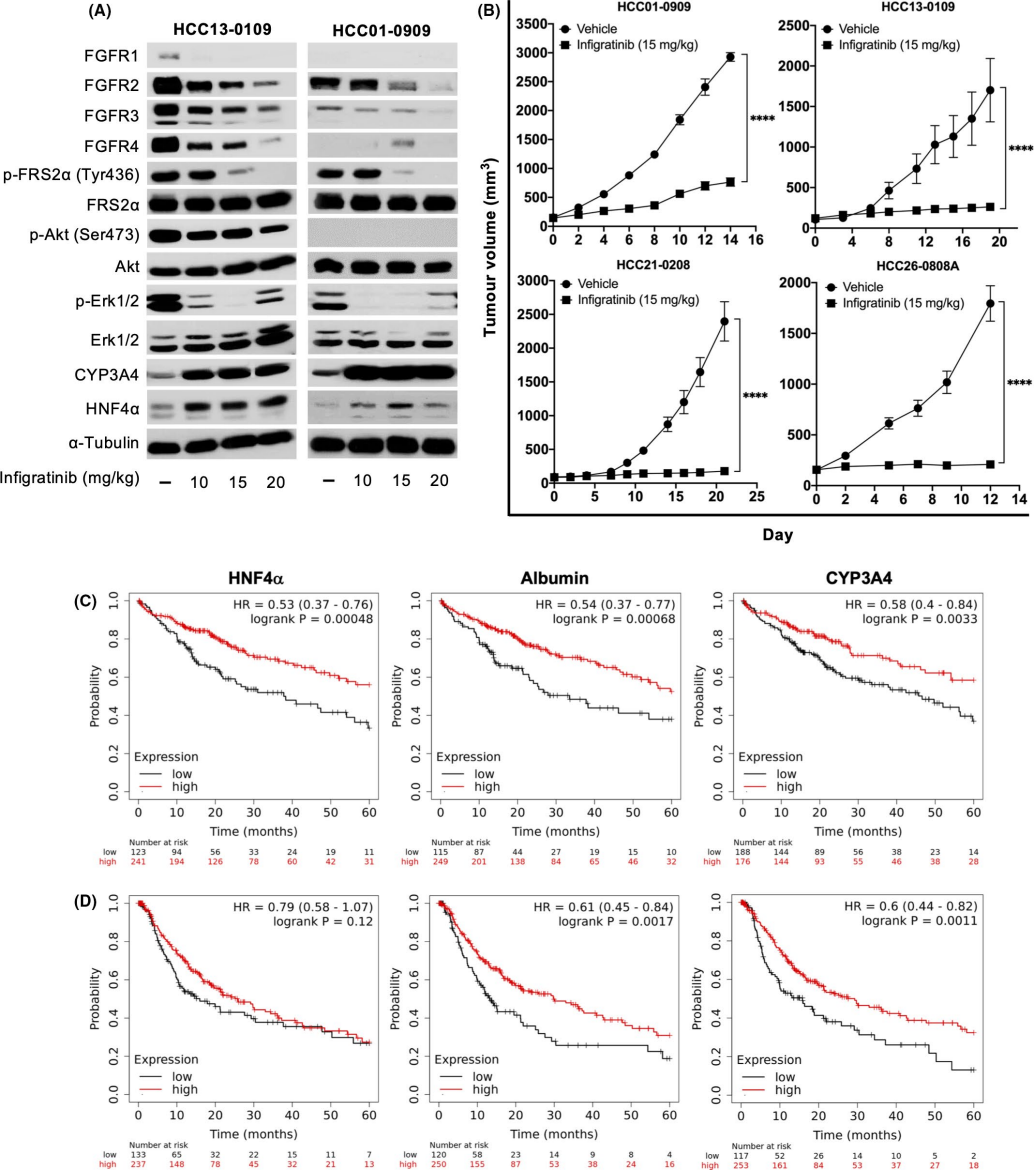
Efficacy of infigratinib in HCC PDX models and its effect on cell proliferation, blood vessel formation and intratumoural hypoxia. To determine the optimal infigratinib dose, mice bearing the indicated tumours (n = 10 per treatment group) were orally administered vehicle or 10, 15 or 20 mg/kg infigratinib for 4 days when tumours reached approximately 800 mm^3^. Tumour lysates were subjected to Western blot analysis and membranes were probed with the indicated antibodies (A). Mice were treated with 15 mg/kg infigratinib once daily when tumours reached roughly 150‐175 mm^3^ and tumour growth was measured over the indicated number of days. Mean volume ± SE is plotted (B). Five‐year OS (C) and PFS (D) in HCC patients expressing high and low levels HNF4α, albumin and CYP3A4. ****P ≤ .0001 (Two‐way ANOVA)

To determine the optimum dose for the induction of differentiation markers, mice bearing HCC13‐0109 and HCC01‐0909 PDX models were administered 10, 15 or 20 mg/kg infigratinib for 4 days. Infigratinib reduced the levels of FGFR‐1, ‐2, ‐3 and ‐4 concomitant with a decrease in its mRNA levels (Figure 1A and Figure S1A). Consistent with our previous study,^31^ 15 mg/kg infigratinib was shown to reduce the expression of FGFR signalling proteins, such as p‐FRS2α, p‐Akt and p‐ERK1/2. Furthermore, infigratinib induced the expression of markers of differentiated cells (CYP3A4 and HNF4α) in a dose‐dependent manner, suggesting its mechanism in promoting cell differentiation (Figure 1A and Figure S1B). Next, the antitumour activity of infigratinib was examined on FGFR‐expressing HCC01‐0909, HCC13‐0109, HCC21‐0208 and HCC26‐0808A PDX models. A daily dose of 15 mg/kg infigratinib significantly reduced tumour growth in all high‐FGFR‐expressing PDX lines (Figure 1B). Similar results were observed in other high FGFR‐expressing PDX lines (Table S1).

Histological analyses suggest that infigratinib inhibits tumour cell proliferation, as indicated by a decrease in the number of cells positively stained for p‐Histone H3 Ser10. Compared with the vehicle‐treated group, infigratinib‐treated mice showed a higher density of morphologically distinct intratumoural blood vessels. Vehicle‐treated mice displayed dysregulated and non‐productive blood vessels associated with the presence of large hypoxic regions. In contrast, infigratinib‐treated tumours showed slim blood vessels resembling normal capillaries and a significant reduction in hypoxic regions via vascular normalization (Figure S1C). These indicate the ability of infigratinib to suppress tumour proliferation and induce tumour microenvironment remodelling.

Conversely, in low‐FGFR‐expressing HCC10‐0505, HCC26‐1004 and HCC2‐1318, infigratinib did not appear to suppress tumour growth, despite the higher dose of infigratinib used (20 mg/kg). Compared with vehicle, p‐Histone H3 Ser10 staining showed similar numbers of staining‐positive cells, indicating the lack of tumour growth inhibition. Furthermore, CD31 staining also exhibited morphologically large and dysregulated blood vessels (Figure S2A‐C).

We then analysed the 5‐year overall survival (364 patients) and progression‐free survival (PFS; 370 patients) of HCC patients based on the expression of the differentiation markers HNF4α, ALB and CYP3A4. Kaplan‐Meier plots were generated based on liver cancer RNA‐seq data from the publicly available KM Plotter (https://kmplot.com/).^35^ High expression of HNF4α, albumin and CYP3A4 correlates with a better 5‐year OS (Figure 1C) and also predicts better PFS (Figure 1D). These highlights the potential clinical significance of infigratinib‐induced cell differentiation, where tumours with higher expression of HNF4α, albumin and CYP3A4 were less proliferative, therefore reducing disease progression and improving overall survival.

### 3.2 Mechanisms of infigratinib‐induced cell differentiation in HCC PDX models

**FIGURE 2:**
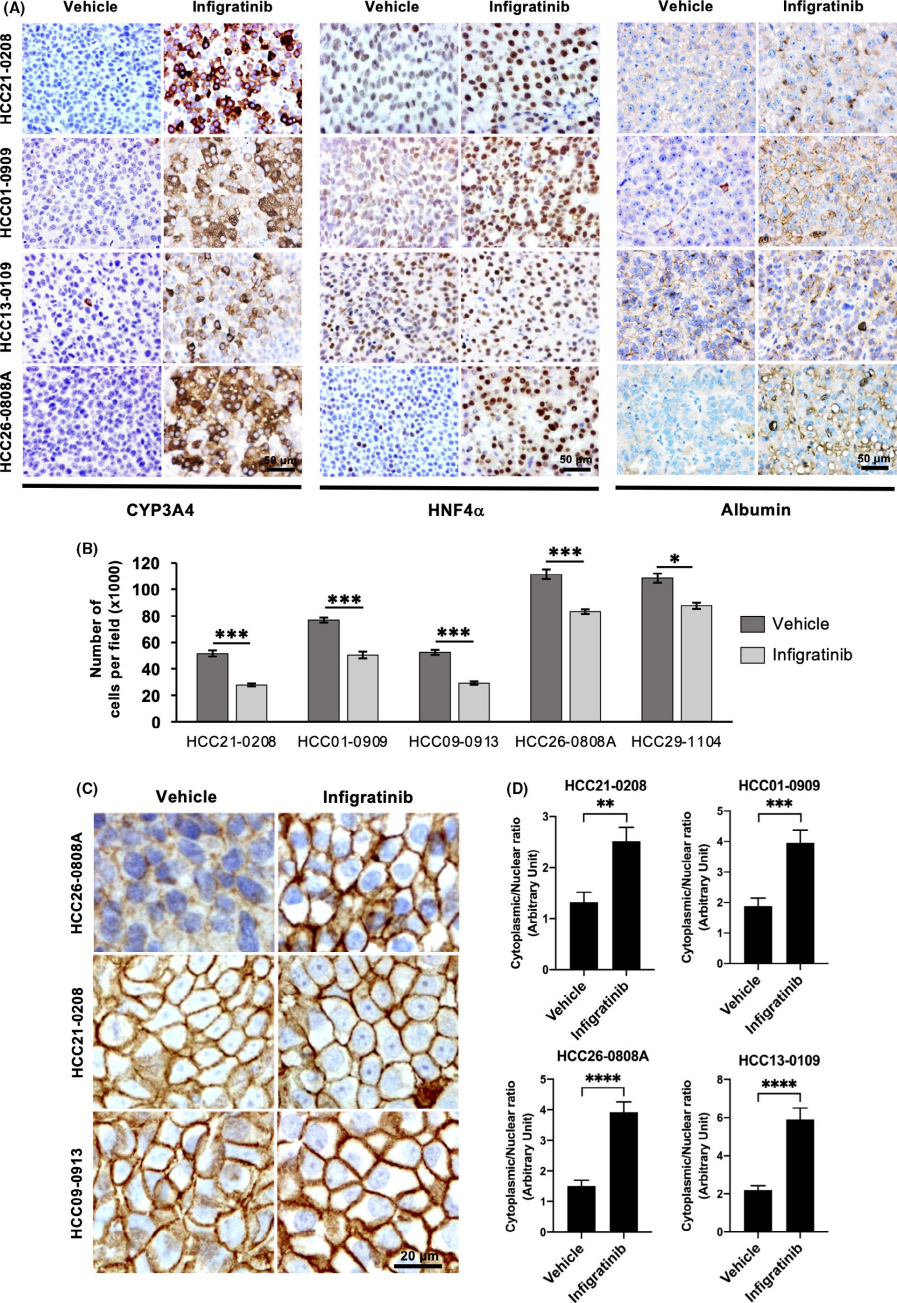
Infigratinib induces cell differentiation in HCC PDX models. Mice bearing the indicated tumours (n = 10 per treatment group) were orally administered vehicle or 15 mg/kg infigratinib for 7 days. Tumour tissues were collected and subjected to histological analysis for markers of hepatocyte differentiation, including CYP3A4, HNF4α and albumin (A); scale bar: 50 µm. The number of cells per field of view was quantified as an indication of morphological changes resulting from cell differentiation (B). Tissue sections were stained with β‐catenin and haematoxylin (C) (scale bar: 20 µm) and the areas of the nucleus and cytoplasm were calculated as described. The ratio of the cytoplasm to nucleus was determined (D) and indicates the extent of cell differentiation. *P ≤ .05; **P ≤ .01; ***P ≤ .001; ****P ≤ .0001 (Student's *t* test)

Next, we sought to determine the molecular mechanism for infigratinib‐induced cell differentiation in HCC PDX models. Tumour sections were stained for markers of hepatocyte differentiation, such as CYP3A4, HNF4α and albumin. Infigratinib‐treated tumours showed higher expression of the differentiation markers CYP3A4, HNF4α and albumin, indicating a reduction in stemness (Figure 2A). Compared with vehicle‐treated tumours, infigratinib‐treated tumours displayed a larger cell morphology, which was evident from the lower number of cells per field of view (Figure 2B). To define boundaries between cells, tumour sections were stained with β‐catenin, and the area ratio of the cytoplasm to the nucleus was quantified. The cytoplasmic/nuclear ratio in infigratinib‐treated tumours was significantly larger, indicating well‐differentiated cells (Figure 2C,D).

In contrast, no morphological changes and no difference in CYP3A4, albumin and HNF4α levels were detected following infigratinib treatment in low‐FGFR‐expressing PDX models. Despite expressing FGFR4, HCC10‐0505 and HCC26‐1004 (Figure S2B) did not appear to undergo cell differentiation, indicating that differentiation is primarily regulated through FGFR1‐3 pathways and that the levels of FGFR can be used as a predictive marker for response to infigratinib treatment (Figure S2D).

### 3.3 Long‐term treatment with infigratinib results in the emergence of resistant colonies

**FIGURE 3:**
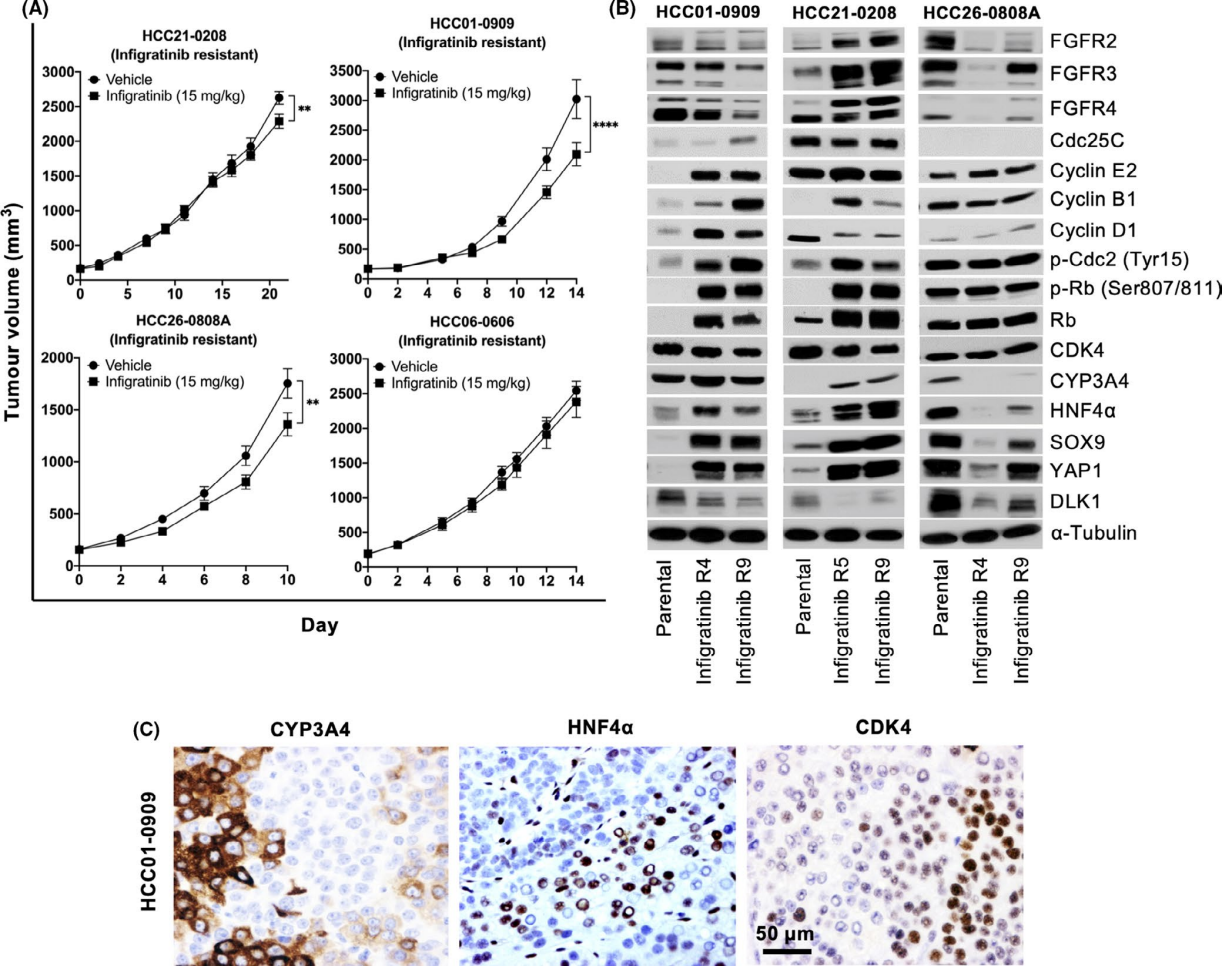
Long‐term treatment with infigratinib results in the emergence of dedifferentiated‐resistant colonies characterised by an increase in cell cycle proteins. Infigratinib‐resistant PDX models were generated by daily administration of 15 mg/kg infigratinib until complete resistance was observed. Resistant PDX models were maintained for several passages and the mean tumour volume ± SE compared with vehicle‐treated tumours (A). Representative blots of tumour lysates collected at different passages. ‘R’ indicates the number of times the tumour was re‐implanted and treated with infigratinib (B). Tumour tissues collected at the early stages of disease progression following long‐term infigratinib treatment were stained with CYP3A4, HNF4α and CDK4 antibodies and representative photographs were shown (C). **P ≤ .01; ****P ≤ .0001 (Two‐way ANOVA). Scale bar: 50 µm

To assess the possible mechanisms responsible for infigratinib resistance, infigratinib‐resistant PDX models were generated by continuous daily dosing of 15 mg/kg infigratinib to tumour‐bearing mice until complete resistance observed. Compared with the antitumour response observed in naïve PDX models (Figure 1C), infigratinib‐treated‐resistant tumours grew at a comparable rate as vehicle‐treated tumours (Figure 3A). Western blot analyses from infigratinib‐resistant tumour collected at different passages indicated an elevation in cell cycle proteins such as cyclin E2, cyclin B1, Cdc25C, p‐Cdc2, cyclin D1, Rb and p‐Rb (Figure 3B). This suggests that resistance to infigratinib is partly acquired through the elevation of cell cycle pathways and that the addition of cell cycle inhibitor might overcome resistance to infigratinib. Interestingly, our PDX models suggest that the expression of cell differentiation markers is maintained, indicating that a portion of resistant tumours still retain the ability to differentiate (Figure 3B).

We then sought to investigate the molecular and cellular transformations that occurred during the early stages of infigratinib resistance. Analyses of tumour sections collected at the early stages of disease progression following infigratinib treatment revealed dedifferentiated cells, as indicated by colonies with distinct cellular organisation in the background of differentiated tumour, whereby cells were more tightly packed, resembling undifferentiated CSCs (Figure 3C). This was further supported by Western blot analyses on infigratinib‐resistant tumours, where there was an increase in CSC markers such as SOX9, YAP1 and DLK1 particularly in the later stages of resistance (Figure 3B). Furthermore, the absence of differentiation markers CYP3A4 and HNF4α was specific to these clusters of resistant cells with low cytoplasmic/nuclear ratios, as the surrounding differentiated cells still retained the expression of those markers and exhibited higher cytoplasmic/nuclear ratios. These resistant nodules also appeared to exhibit an increase in CDK4 expression (Figure 3C), consistent with the Western blot analyses of resistant PDX tumour (Figure 3B). A similar result was observed in other PDX line (Figure S3A).

### 3.4 Ribociclib reverses resistance to infigratinib and results in more complete cell differentiation

**FIGURE 4:**
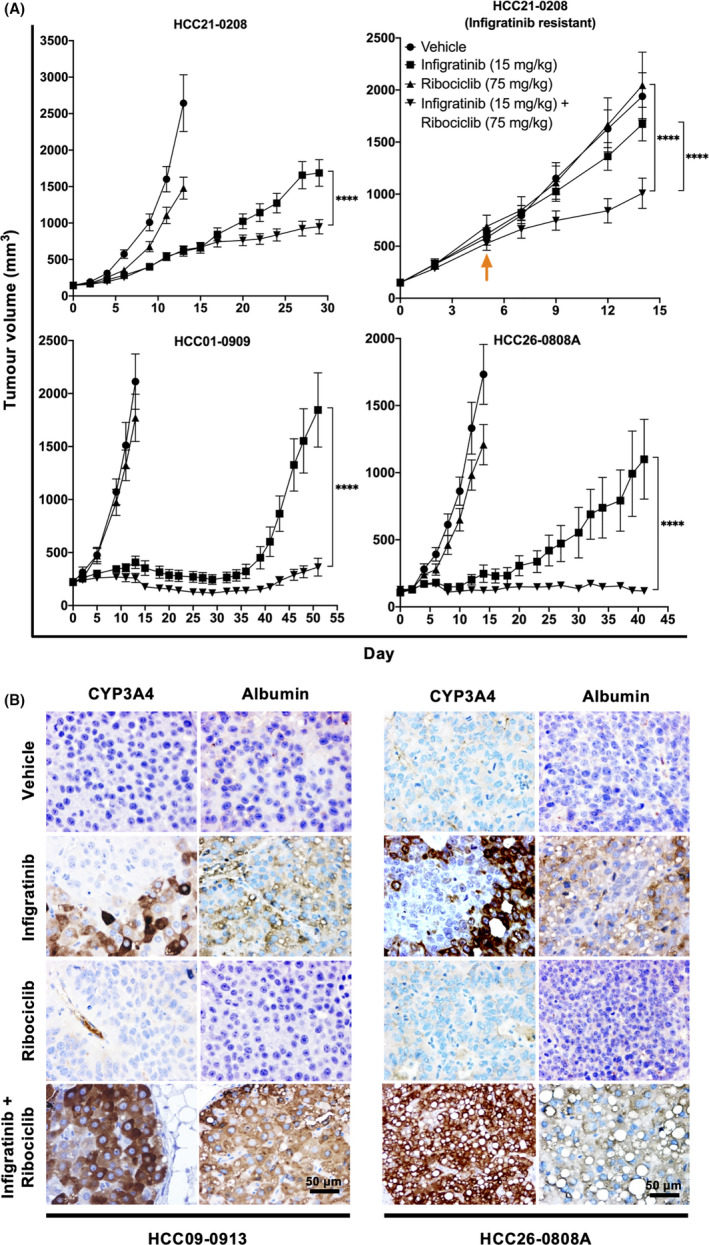
Ribociclib and infigratinib induces longer‐lasting cell differentiation. Mice bearing the indicated tumours were orally administered vehicle, 15 mg/kg infigratinib, 75 mg/kg ribociclib or a combination of 15 mg/kg infigratinib and 75 mg/kg ribociclib (n = 10 per group). Tumour volumes were measured over time, and the mean volume ± SE is plotted (A). For the combination therapy in infigratinib‐resistant HCC21‐0208, ribociclib was introduced on day 5 (indicated by an arrow). Tumour sections were stained with the cell differentiation markers CYP3A4 and albumin. The addition of ribociclib abrogated infigratinib‐resistant colonies and promoted more differentiated cells, as indicated by CYP3A4 and albumin staining (B). ****P ≤ .0001 (Two‐way ANOVA). Scale bar: 50 µm

Since infigratinib resistance appeared to be mediated by an increase in cell cycle proteins (Figure 3B), we investigated the possibility of adding the cell cycle inhibitor ribociclib into the treatment regimen. A daily dose of 75 mg/kg ribociclib alone only modestly inhibited tumour growth in HCC21‐0208, HCC01‐0909 and HCC26‐0808A. Despite showing high levels of cell cycle‐related proteins in the parental tumour (Figure 3B), HCC26‐0808A did not appear to respond to ribociclib inhibition (Figure 4A). This is presumably because of its high dependence on FGFRs.

The addition of ribociclib significantly prolonged the antitumour effect of infigratinib in HCC01‐0909, HCC21‐0208 and HCC26‐0808A PDX models. For example HCC01‐0909 cells treated with infigratinib alone began to show resistance at around day 36, followed by an exponential tumour progression in subsequent days. In contrast, mice treated with the combination of infigratinib and ribociclib showed a significantly prolonged antitumour effect until the end of treatment (day 51). These results correspond to the smaller tumour weight observed at the end of the treatment cycle (Figure S4A). Similar results were also observed in other PDX lines investigated (Figure S4B). Furthermore, infigratinib‐resistant HCC21‐0208 treated with the combination infigratinib/ribociclib also showed significant inhibition of tumour growth (Figure 4A). Importantly, the antitumour effect of ribociclib/infigratinib was observed even when ribociclib was introduced after tumour reached approximately 600 mm^3^. This provides a more realistic clinical model, where ribociclib is likely to be introduced when progression was observed following infigratinib treatment.

Immunohistochemical analyses showed an absence of resistant colonies and an increase in CYP3A4 and albumin expression in drug combination‐treated tumours. The majority of tumour cells in the drug combination‐treated tumours had morphologic evidence of the terminal hepatocyte differentiation and exhibited a uniformly higher expression of CYP3A4 and albumin staining throughout the tissue section (Figure 4B; Figure S3B). This suggested that the drug combination significantly reduced the emergence of resistant nodules, where it was observed in roughly half of the infigratinib‐treated tumours.

**FIGURE 5:**
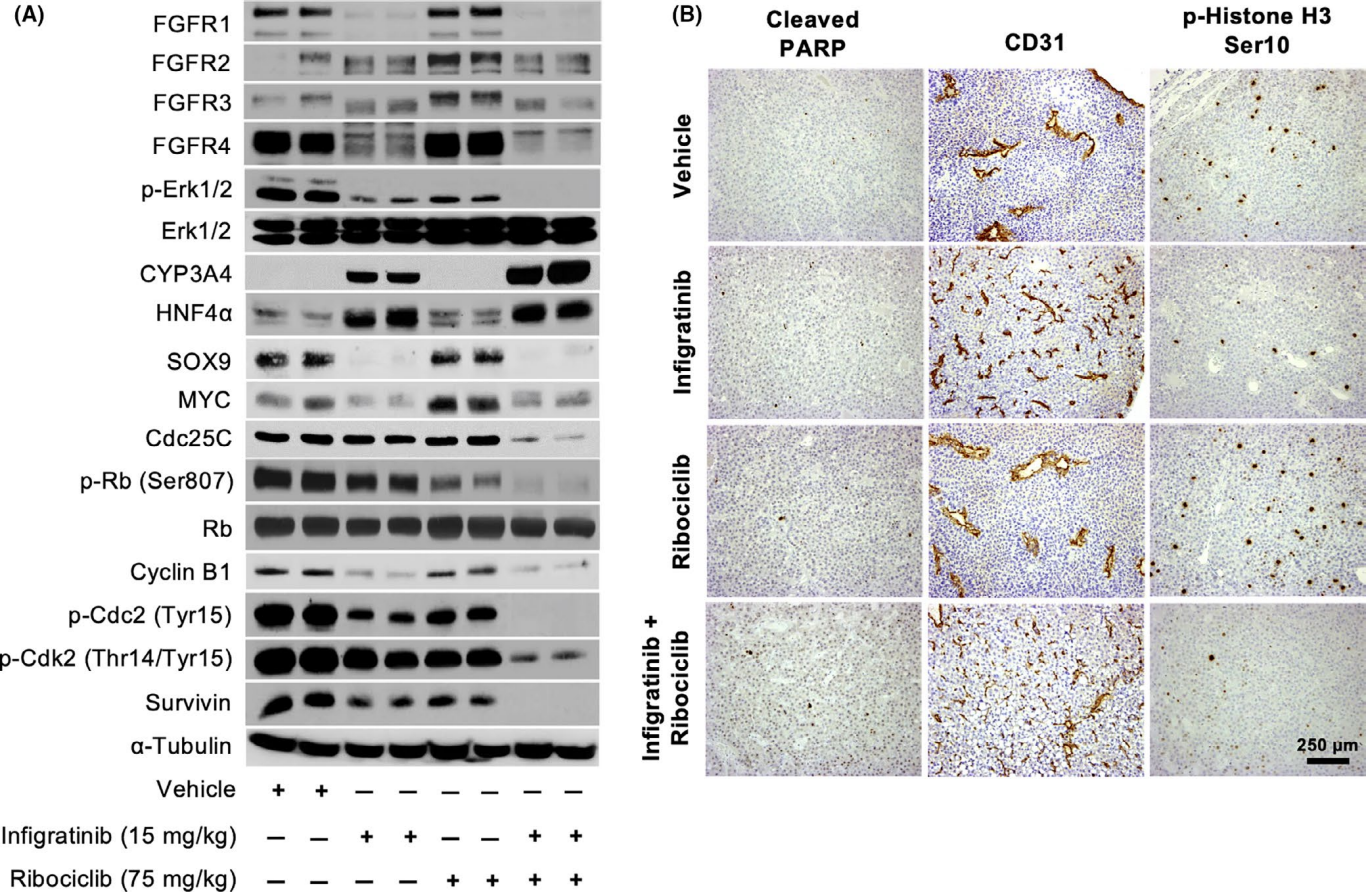
The effects of ribociclib and infigratinib on the FGFR/CDK4/6/pRb pathway, and on cell proliferation and intratumoral blood vessel. Mice bearing HCC21‐0208 PDX were treated for 4 days and tumour lysate subjected to Western blot analyses. Treatments started when the tumours were approximately 800 mm^3^. Membranes were probed with the indicated antibodies and representative blots are shown (A). Sections from the same tumours were stained with cleaved PARP, CD31 and p‐Histone H3 Ser10 and representative photographs shown (B). Ribociclib and infigratinib increased blood vessel density and inhibited cell proliferation. Scale bar: 250 µm

Western blot analyses further indicated that treatment with 75 mg/kg ribociclib alone only partially suppress cell cycle proteins such as Cdc25C, cyclin B1 and p‐Cdc2 in HCC21‐0208 tumour. Furthermore, no increase in CYP3A4 and HNF4α expression was detected (Figure 5A), consistent with immunohistochemical analyses (Figure 4B). Similar expression patterns were observed in HCC13‐0109 and HCC01‐0909 tumours (Figure S5A). Interestingly, ribociclib treatment also appeared to slightly increase the expression of FGFRs, suggesting a feedback loop mechanism (Figure 5A; Figure S5A). However, when combined, infigratinib and ribociclib synergistically abrogated the expression of proteins involved in proliferation, survival and stemness, such as SOX9 and MYC, effectively reversing resistance to infigratinib and allowing cells to remain in the differentiated state. Moreover, infigratinib/ribociclib treatment was more potent than infigratinib alone in inducing HNF4α and CYP3A4 expression and suppressing cell cycle proteins such as Cdc25C, survivin, cyclin B1 and p‐Cdc2 (Figure 5A; Figure S5A).

While infigratinib treatment alone was able to normalise blood vessels and suppress tumour proliferation, the combination treatment showed an even higher density of blood vessels, as evidenced by CD31 staining, and less proliferative cells, as indicated by the reduction in cells positively staining for p‐Histone H3 Ser10 (Figure 5B; Figure S5B). Collectively, these results describe the synergistic mechanism of infigratinib and ribociclib in inducing long‐term differentiation in HCC PDX models. Importantly, the combination of 15 mg/kg infigratinib and 75 mg/kg ribociclib did not appear to induce acute toxicity, as indicated by the body weight of mice at the end of treatment (Figure S6).

## 4 DISCUSSION

Hepatocellular carcinoma remains one of the leading causes of cancer‐related mortality worldwide, yet there has been no significant improvement in systemic treatment options in nearly two decades.^36^ Advancements in radiotherapy have somewhat improved treatment outcomes, but this is mostly limited to localised tumours.^37^ Targeting cancer stem cells to induce terminal differentiation has gained interest in recent years, following the success of a similar treatment strategy in leukaemia.^38^, ^39^ In this study, we examined the potential of infigratinib to induce HCC differentiation and explored the potential of combining treatment with ribociclib to reduce drug resistance and achieve a long‐lasting response.

The FGFRs are members of the tyrosine kinase family, and their dysregulations have been shown to play a key role in liver carcinogenesis.^40^, ^41^, ^42^ It has been reported that the levels of FGFR2 and FGFR3 strongly correlate with poor tumour differentiation.^40^, ^43^ Consistent with our previous study, treatment of FGFR‐expressing HCC models with infigratinib resulted in inhibition of FGFR activity, suppression of tumour growth, inhibition of cell proliferation, induction of vascular normalisation and reduction in tumour hypoxia (Figure 1). Since overexpression of the FGFR family has been associated with increased stemness,^42^, ^44^ we sought to determine whether blockade of this signalling pathway leads to cell differentiation. We demonstrated that infigratinib potently induced HCC cell differentiation in an FGFR‐dependent fashion. Tumours lost their neoplastic histological features, such as a high mitotic index, large nucleoli and hyperchromasia and exhibited a significant increase in the cytoplasmic/nuclear ratio. The differentiated tumour cells were p‐Histone 3 Ser10‐negative, consistent with their reduced rate of cellular proliferation. All differentiation markers (CYP3A4, HNF4α and albumin) in infigratinib‐treated FGFR‐dependent tumours were significantly increased compared with their levels in vehicle‐treated tumours (Figure 2A). This indicates that tumour cells are able revert to a state of dormancy.^45^, ^46^ Clinically, HCC patients with higher levels of CYP3A4, HNF4α and albumin showed better 5‐year OS and PFS compared with those patients exhibiting lower levels of these differentiation markers (Figure 1C,D), supporting the potential of infigratinib‐mediated differentiation therapy in HCC.

The concept of differentiation therapy pivots towards keeping cancer cells in their dormant state rather than eradicating them completely. Therefore, we investigated the feasibility of long‐term infigratinib treatment. Despite showing an initial response, we observed the emergence of resistant colonies after prolonged treatment with infigratinib, consistent with previous reports.^11^, ^15^, ^16^ However, the molecular mechanisms responsible for the acquired resistance to infigratinib have not been defined. In this study, we observed that these resistant cells showed an upregulation of proteins involved in various stages of the cell cycle, such as Cdc25C, cyclin E2, cyclin B1, cyclin D1 and p‐Cdc2. This was also associated with the upregulation of Rb and p‐Rb (Figure 3). The cyclin D1‐CDK4/6 complex, together with the cyclin E2‐Cdk2 complex and hyperphosphorylated p‐Rb subsequently releases the E2F transcription factor, ultimately driving the expression of genes involved in the S phase.^47^ Notably, a subpopulation of these resistant tumours still retains the ability to differentiate, as indicated by the expression of HNF4α, suggesting that cellular differentiation is a plastic process as opposed to one that is unidirectional.^48^

On the basis of this observation, we hypothesised that inhibition of the cell cycle pathway using ribociclib could overcome infigratinib resistance. Clinically, ribociclib is an inhibitor of the cyclin D1/CDK4/6, which is commonly used in the treatment of hormone‐receptor‐positive breast cancer.^49^ Indeed, the addition of ribociclib promotes tumour cell differentiation, reverses resistance to infigratinib and significantly prolongs the cytostatic effect of infigratinib (Figure 4). Furthermore, no evidence of toxicity was observed in any animals treated with the drug combination, as indicated by the similar body weights across all treatment groups (Figure S6).

Mechanistically, ribociclib treatment also appears to slightly upregulate FGFR in some HCC lines, suggesting the presence of a feedback mechanism that forces cells to depend on the FGFR pathway to continue to proliferate (Figure 5A; Figure S5A). Immunohistochemical analyses revealed that cells treated with the combination of ribociclib and infigratinib showed more complete cell differentiation, as evidenced by the increased expression of differentiation markers such as CYP3A4 and albumin. Furthermore, staining was found to be more uniform and virtually no resistant nodules were detected (Figure 4B). In addition, the number of p‐Histone H3 Ser10‐positive cells was markedly decreased (Figure 5B), consistent with the notion that terminally differentiated cells were less proliferative.^50^ Infigratinib treatment alone has been shown to normalise intratumoural blood vessels, and this effect was maintained when ribociclib was added. However, ribociclib treatment alone did not show this blood vessel remodelling effect. This suggests that the synergistic effect between infigratinib and ribociclib could partially be explained by the increased delivery of ribociclib to the tumour, facilitated by a more functional blood vessel network (Figure 5B).

**FIGURE 6:**
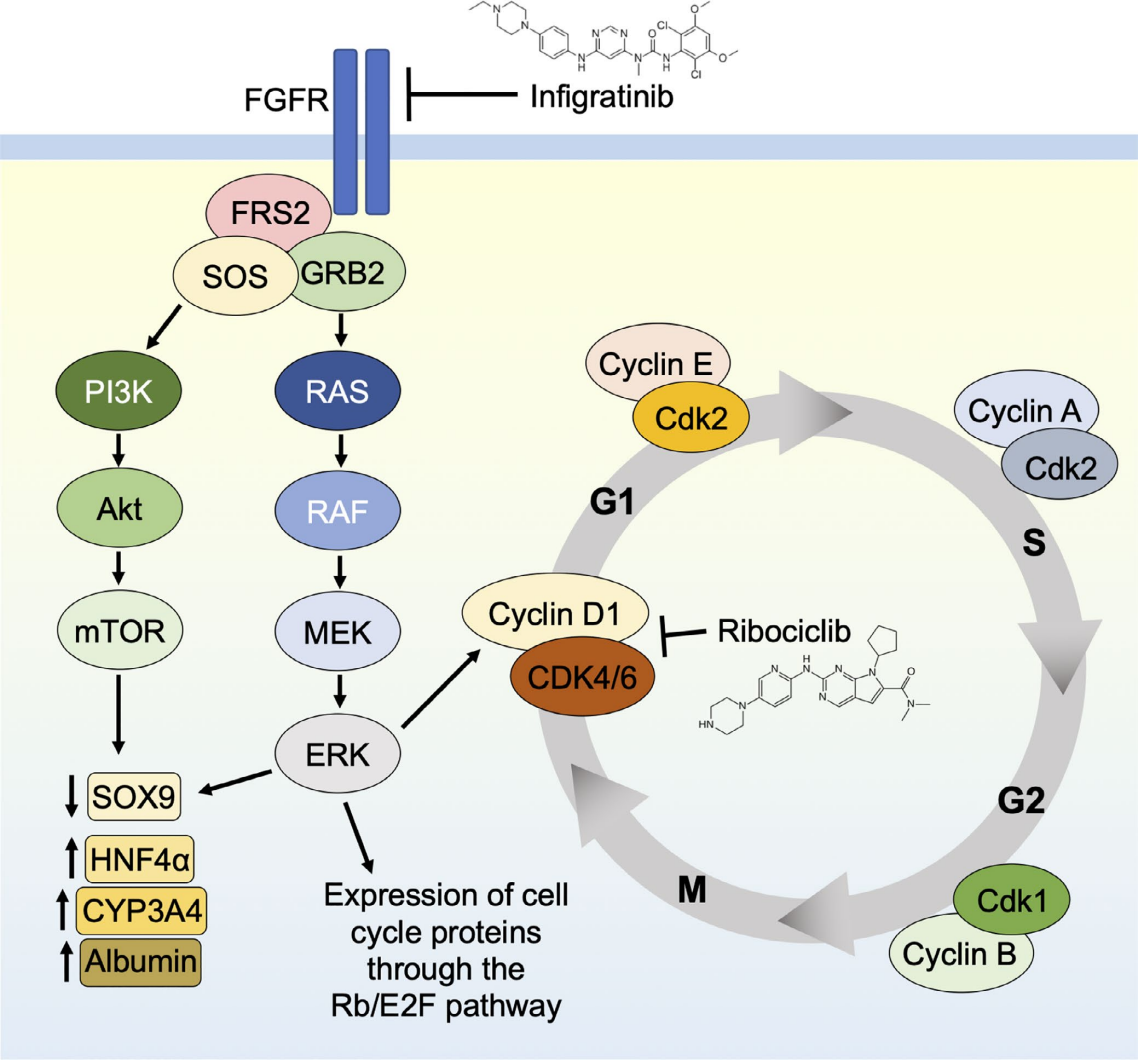
Proposed mechanism of infigratinib and ribociclib in inducing differentiation and preventing dedifferentiation. Infigratinib inhibits FGFRs and effectively blocks downstream Ras/Raf/Mek and PI3K/Akt/mTOR pathways. This may lead to cell differentiation by reducing the expression of stem‐cell‐associated genes through the suppression of transcription factors such as SOX9. In addition, infigratinib reduces the expression of cell cycle proteins through the reduction in the Rb/E2F pathway. As cells gain resistance to infigratinib, cell cycle proteins are upregulated, leading to the inactivation of Rb and cell cycle progression. Ribociclib reverses resistance by blocking the CDK4/6/pRb axis, resulting in an increase in CYP3A4, albumin and HNF4α expression, forcing cells to remain in a differentiated state

Taken together, our data suggest that the inhibition of FGFR1‐3 by infigratinib resulted in the induction of cell differentiation and therefore a reduction in cell proliferation. Canonically, blockade of FGFR signalling led to the attenuation of the Ras/Raf/Mek/Erk and PI3K/Akt/mTOR pathways, leading to a decrease in the expression of proteins involved in cancer stemness. As hepatocytes gained resistance to infigratinib, they displayed a dedifferentiated morphology resembling those of cancer stem cells. These cells were characterised by a loss of differentiation markers and an increase in proteins involved in the cell cycle, such CDK4, leading to the inactivation of Rb. However, this was mitigated by the addition of ribociclib, where the combination of both drugs was able to synergistically block compensatory signalling mechanisms, resulting in longer‐lasting differentiation into mature hepatocytes and a significant reduction in drug resistance (Figure 6). These findings highlight that the restoration of differentiated phenotypes in HCC cells is imperative for tumour suppression. A recent phase I clinical trial of infigratinib has shown favourable outcome,^51^ therefore our study warrants further clinical investigations to determine whether patients may achieve greater benefit from upfront combination of infigratinib and ribociclib treatment.

## CONFLICT OF INTEREST

None declared.

## Supporting information

Fig S1

Fig S2

Fig S3

Fig S4

Fig S5

Fig S6

Table S1
