# Supplementary figures and images for: Ribociclib enhances infigratinib‐induced cancer cell differentiation and delays resistance in FGFR‐driven hepatocellular carcinoma
Source: Liver Int. 2020 Nov 23;41(3):608–20. doi: 10.1111/liv.14728 (PMC7894323; doi:10.1111/liv.14728)

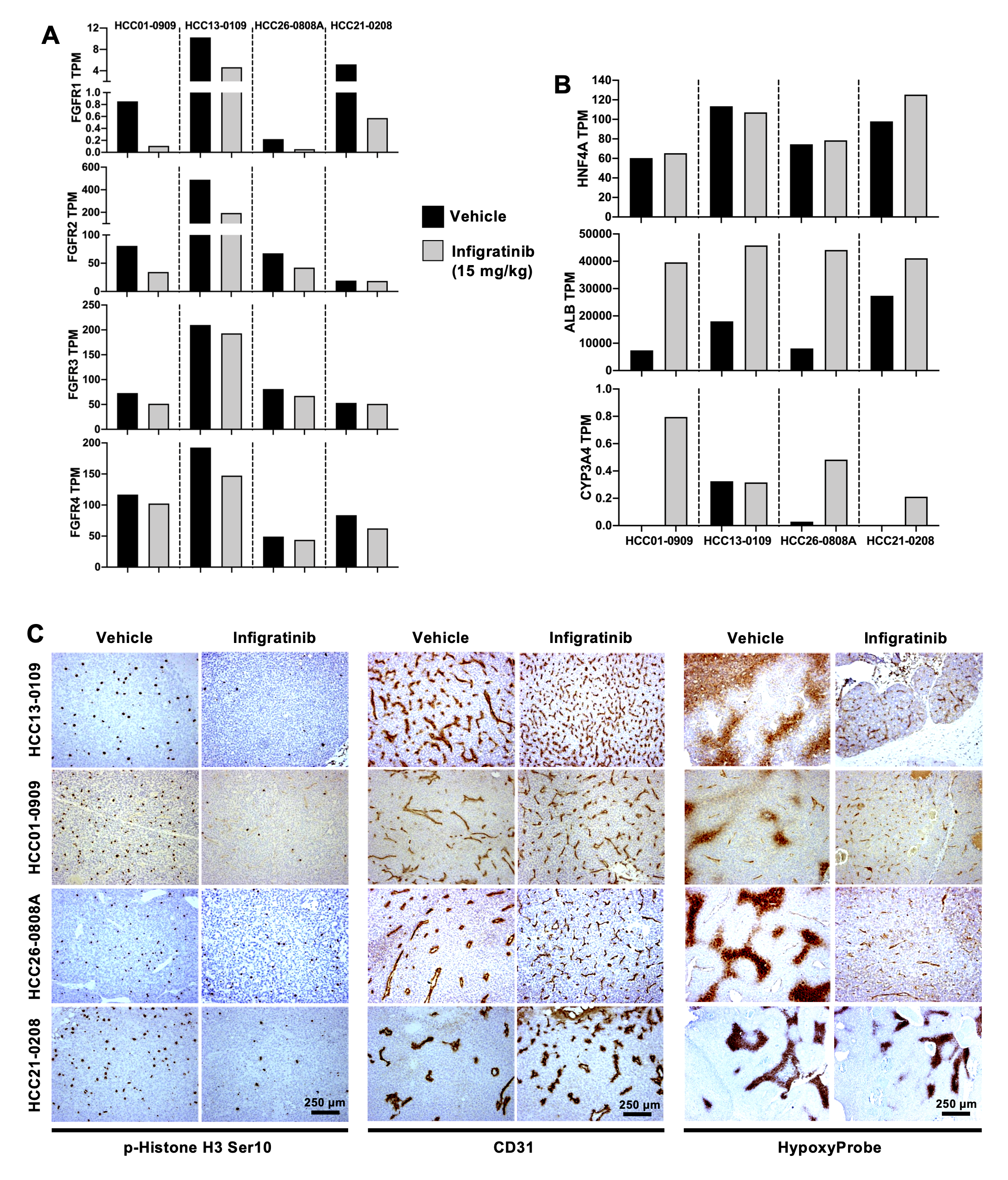

Supplement: Supplementary file 1 — Fig S1 [file LIV-41-608-s001.tiff]

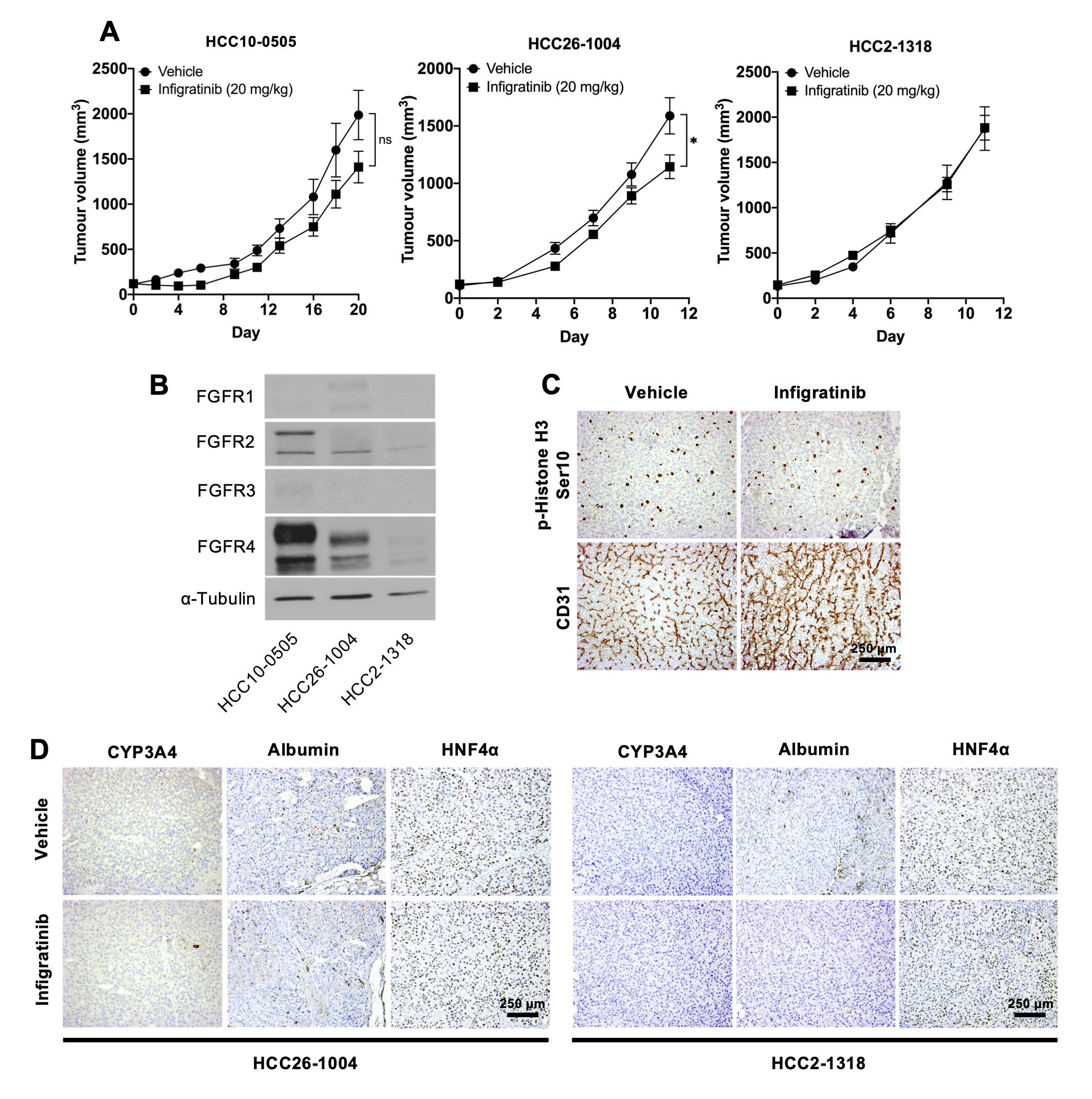

Supplement: Supplementary file 2 — Fig S2 [file LIV-41-608-s002.tiff]

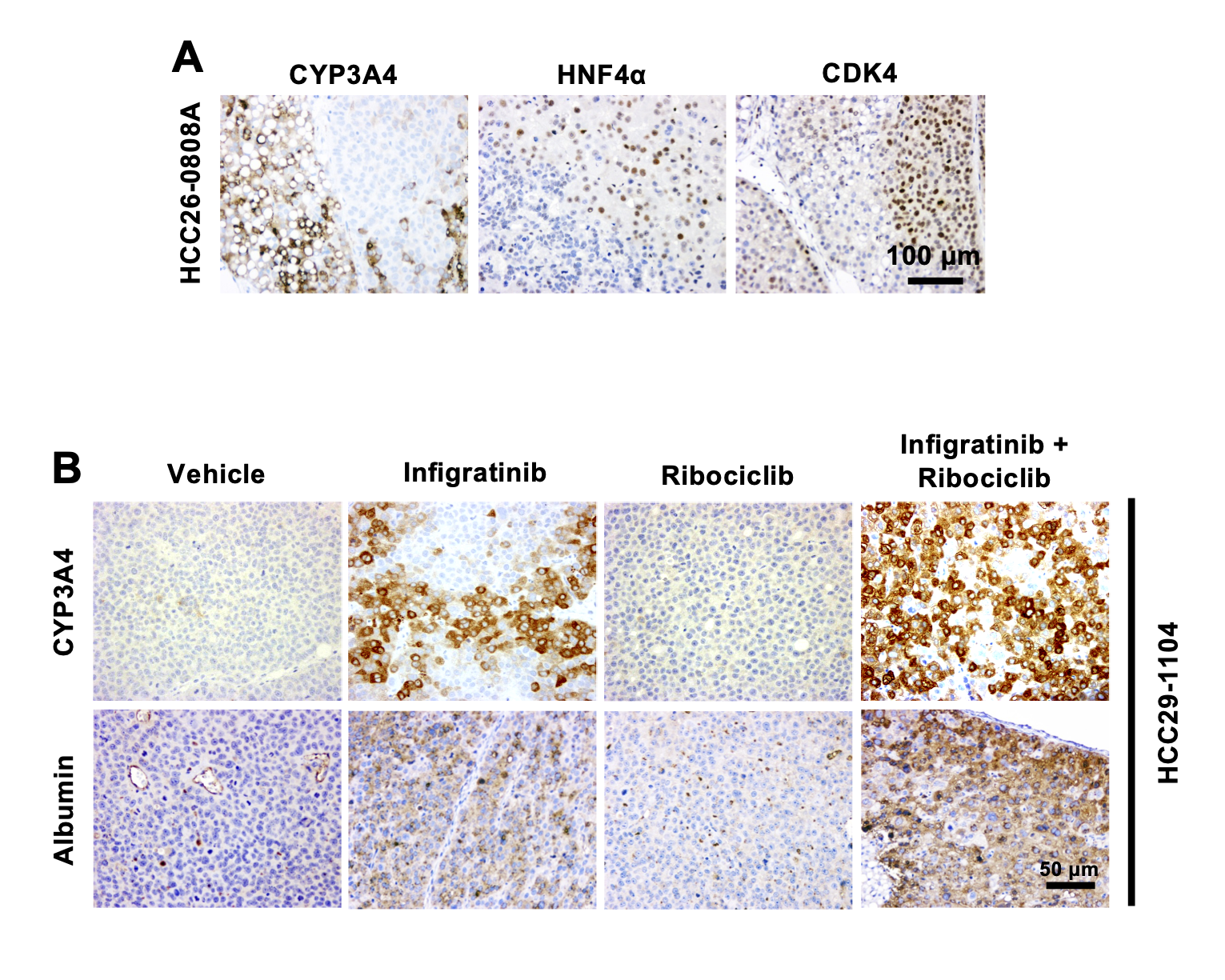

Supplement: Supplementary file 3 — Fig S3 [file LIV-41-608-s003.tiff]

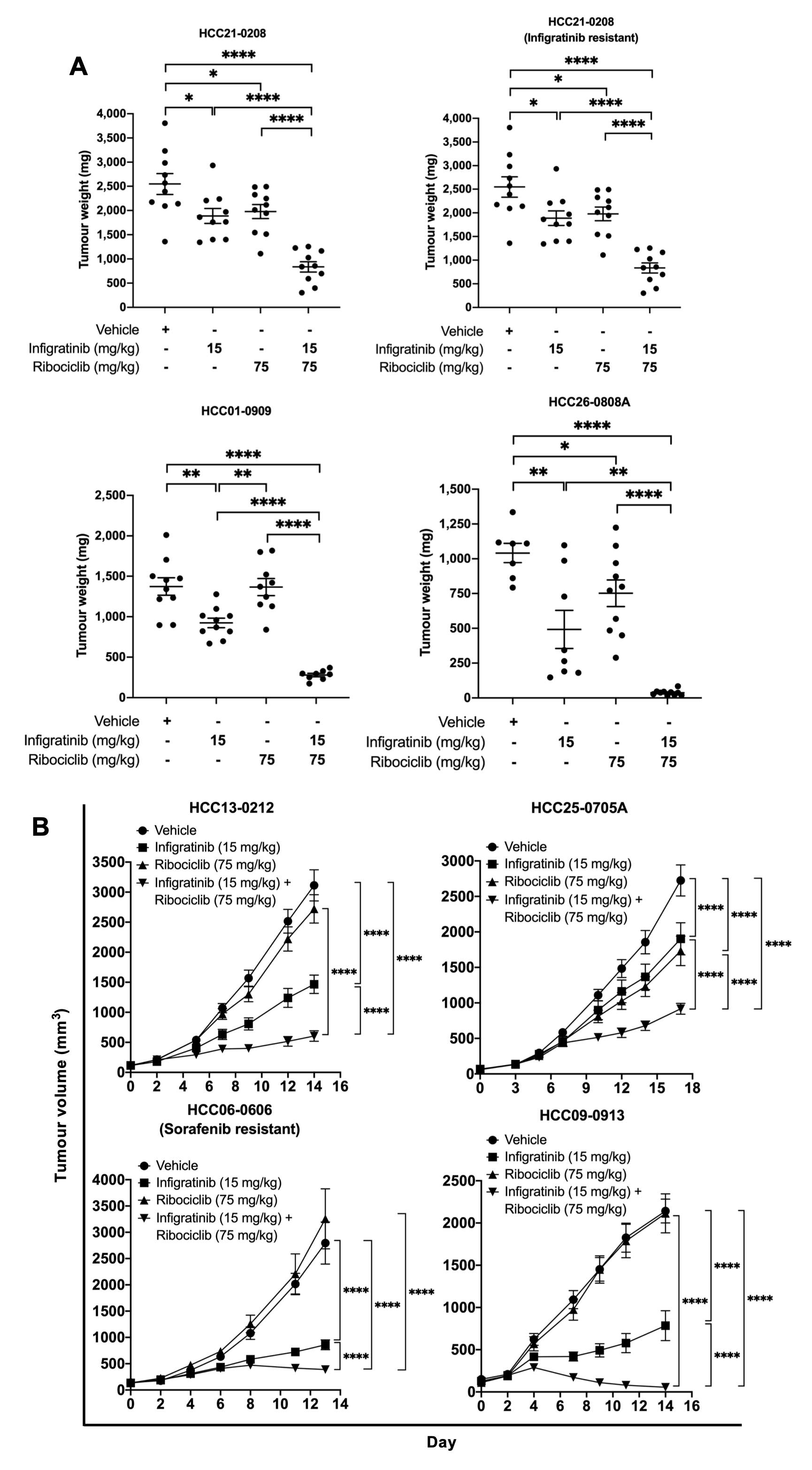

Supplement: Supplementary file 4 — Fig S4 [file LIV-41-608-s004.tiff]

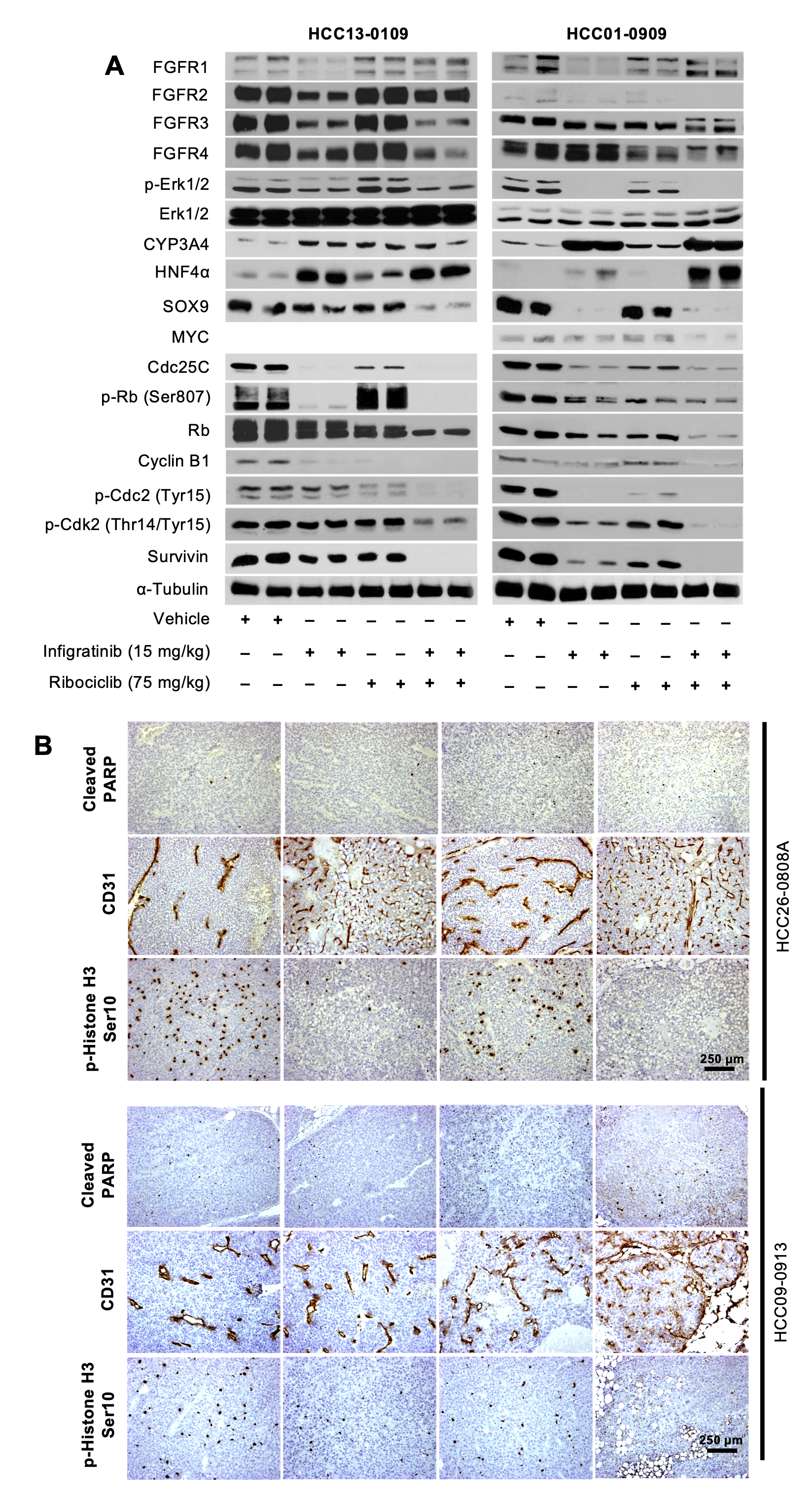

Supplement: Supplementary file 5 — Fig S5 [file LIV-41-608-s005.tiff]

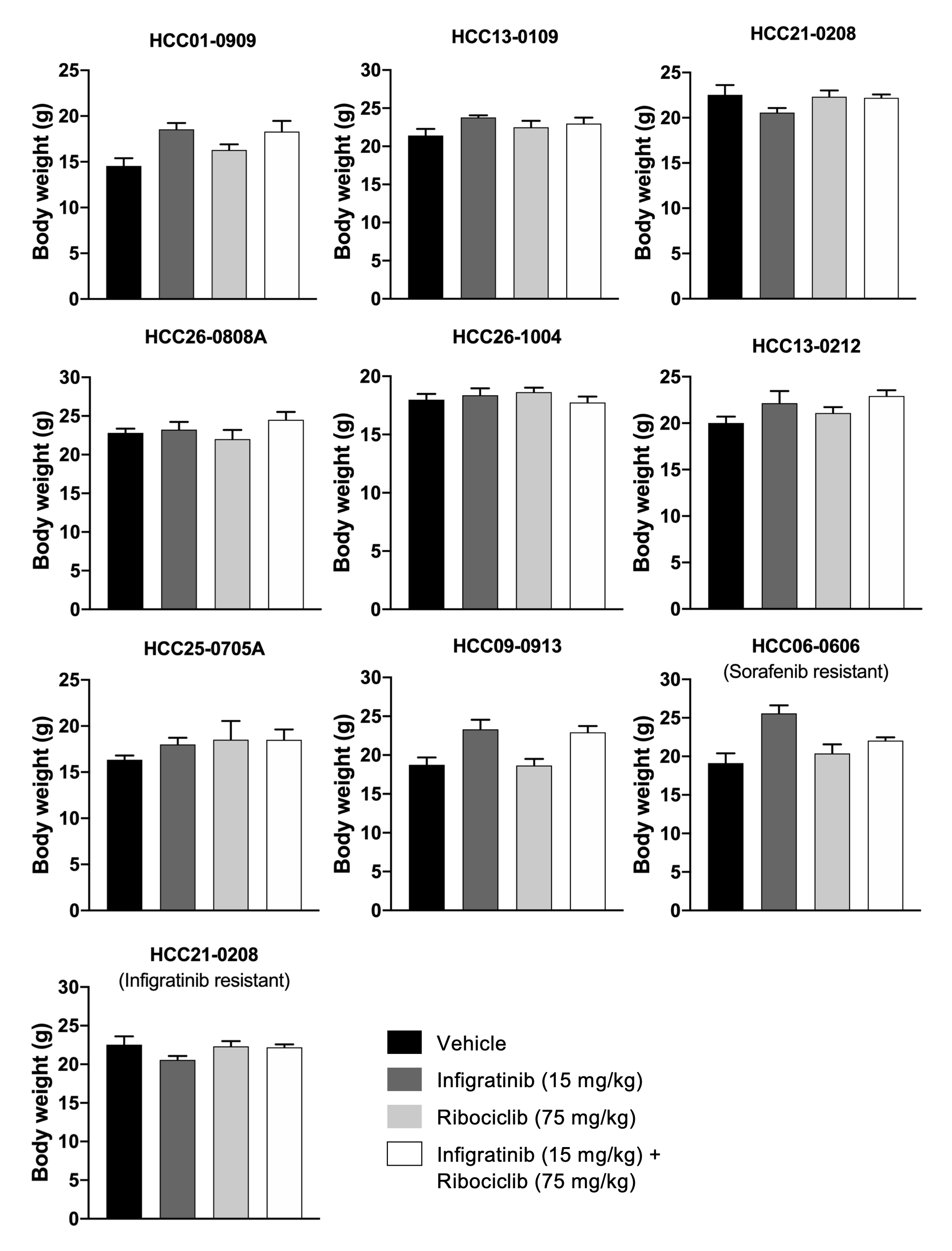

Supplement: Supplementary file 6 — Fig S6 [file LIV-41-608-s006.tiff]
