# Supplementary material for: Ribociclib enhances infigratinib‐induced cancer cell differentiation and delays resistance in FGFR‐driven hepatocellular carcinoma
Source: Liver Int. 2020 Nov 23;41(3):608–20. doi: 10.1111/liv.14728 (PMC7894323; doi:10.1111/liv.14728)
Supplement: Supplementary file 7 — Table S1 [file LIV-41-608-s007.docx]

**Supplementary Table 1.** T/C ratio of HCC PDX models treated with 15 mg/kg infigratinib. SCID mice were subcutaneously implanted with the indicated PDX models and treated with 15 mg/kg infigratinib for 12 to 16 days (depending on the PDX model). Tumour volumes at the end of treatment cycle were normalised to vehicle-treated tumours and expressed as the T/C ratio. A T/C ratio of 0.42 is considered active. * *p* ≤ 0.05; ** *p* ≤ 0.01; *** *p* ≤ 0.001; **** *p* ≤ 0.0001 (Student’s t-test).

| **Xenograft Line** | **T/C ratio** |
| --- | --- |
| HCC21-0208 | 0.47** |
| HCC13-0109 | 0.14**** |
| HCC13-0212 | 0.47** |
| HCC25-0809 | 0.70* |
| HCC06-0606 | 0.28*** |
| HCC09-0913 | 0.25*** |
| HCC26-0808A Sorafenib-resistant | 0.67* |
| HCC06-0606 Sorafenib-resistant | 0.51** |
| HCC26-0808A | 0.16**** |
| HCC25-0705A | 0.47** |
| HCC17-0211 | 0.43** |
| HCC01-0909 | 0.30*** |
| HCC29-1104 | 0.46** |
| HCC02-0113 | 0.67* |
| HCC24-0714 | 0.85 |
| HCC16-1014 | 0.61* |
| HCC19-0913 | 0.96 |
| HCC01-1214 | 0.99 |
| HCC29-0714B | 0.83 |
| HCC11-0314 | 0.83 |
